# Insect-specific Alphamesonivirus-1 (*Mesoniviridae*) in lymph node and lung tissues from two horses with acute respiratory syndrome

**DOI:** 10.1128/jvi.02144-24

**Published:** 2025-01-24

**Authors:** Lucija Jurisic, Heidi Auerswald, Maurilia Marcacci, Francesca Di Giallonardo, Laureen M. Coetzee, Valentina Curini, Daniela Averaimo, Ayda Susana Ortiz-Baez, Cesare Cammà, Giovanni Di Teodoro, Juergen A. Richt, Edward C. Holmes, Alessio Lorusso

**Affiliations:** 1 Istituto Zooprofilattico Sperimentale dell'Abruzzo e del Molise 83371 , Teramo, Italy; 2 Faculty of Veterinary Medicine, University of Teramo 66187 , Teramo, Italy; 3 Faculty of Medicine, The Kirby Institute, University of New South Wales 98994 , Sydney, Australia; 4 School of Veterinary Medicine, Faculty of Health Sciences and Veterinary Medicine, Neudamm Campus, University of Namibia 99404 , Windhoek, Namibia; 5 School of Medical Sciences, The University of Sydney 216920 , Sydney, Australia; 6 College of Veterinary Medicine, Kansas State University 70725 , Manhattan, Kansas, USA; Wageningen University & Research, Wageningen, Netherlands

**Keywords:** Alphamesonivirus-1, *Mesoniviridae*, horses, acute respiratory syndrome, lymph node, lung, metatranscriptomic

## Abstract

Members of the RNA virus order *Nidovirales* infect hosts ranging from marine invertebrates to terrestrial mammals. As such, understanding the determinants of host range in this group of viruses, as well as their patterns of emergence and disease potential, is of clear importance. The *Mesoniviridae* are a recently documented family within the *Nidovirales*. To date, mesoniviruses have only been associated with the infection of arthropod species, particularly mosquitoes, and hence are regarded as insect-specific viruses (ISVs). Herein, we report the first detection of a mesonivirus—Alphamesonivirus-1 —in mammals. Specifically, we utilized genomic and histological techniques to identify Alphamesonivirus-1 in lung and lymph node tissues of two horses (a mare and its foal) from Italy that succumbed to an acute respiratory syndrome. The genome sequences of Alphamesonivirus-1 obtained from the two horses were closely related to each other and to those from a local *Culex* mosquito pool and an Alphamesonivirus-1 previously identified in Italy, indicative of ongoing local transmission. The discovery of Alphamesonivirus-1 in horse tissues prompts further investigation into the host range of mesoniviruses, the possible role of insect-specific viruses in mammalian disease processes, the determinants of and barriers to cross-species virus transmission, and the potential epizootic threats posed by understudied viral families.

**IMPORTANCE:**

Alphamesoniviruses, members of the family *Mesoniviridaeare,* are considered insect-specific RNA viruses with no known association with vertebrate hosts. Herein, we report the identification of Alphamesonivirus-1 in mammals. Using detailed molecular and histological analyses, we identified Alphamesonivirus-1 in lung and lymph node tissues of two horses that presented with an acute respiratory syndrome and that was phylogenetically related to virus sequences found in local *Culex* mosquitoes. Hence, Alphamesoniviruses may possess a broader host range than previously believed, prompting the investigation of their possible role in mammalian disease. This work highlights the need for increased surveillance of atypical viruses in association with unexplained respiratory illness, including those commonly assumed to be insect-specific, and may have implications for epizootic disease emergence.

## INTRODUCTION

Revealing the nature of host-pathogen interactions, including the pathways and barriers to successful cross-species transmission, is central to understanding disease emergence (1). Our knowledge of the host range of many viruses has been greatly enhanced by the increasing use of metagenomic sequencing, which enables the entire viromes of species to be documented. In addition, metagenomic sequencing has advanced diagnostics, enabling the rapid identification and characterization of poorly described animal pathogens. As a case in point, metagenomics has led to a new appreciation of the diversity of RNA viruses within the order *Nidovirales* (single-stranded, positive-sense RNA viruses) (2). However, despite the discovery of a multitude of novel nidoviruses, much of our knowledge of the fundamental biology of this group stems from studies of mammalian coronaviruses, including SARS-CoV, SARS-CoV-2, and MERS-CoV. Indeed, there has been a general neglect of the other families within the *Nidovirales*, including their prevalence, host range, transmission, and capacity to cause disease.

Respiratory problems are common in horses and are often diagnosed as a cause of poor athletic performance. However, the basic diagnostic techniques of the equine respiratory tract examination are not always sufficient for a complete diagnosis of the disease, its exacerbation, remission, or response to treatment. Infections are the most common cause of respiratory system problems in horses. This is particularly so with racehorses, in which respiratory system infections are often cited as the second most common reason for horses failing to train (3). Pathogens of the greatest concern in horses are influenza A viruses (AIV), Equine herpesvirus 1 and 4 (EHV1, EHV4), *Streptococcus zooepidemicus*, *Streptococcus pneumoniae*, *Streptococcus equi* subsp. *equi*, *Rhodococcus equi*, and *Pasteurella* spp. (4–6). In addition, horses are susceptible to a plethora of viruses transmitted by biting arthropods, including mosquitoes, midgets, flies, ticks, as well as parasites (7).

The *Mesoniviridae* are a newly assigned family of viruses within the *Nidovirales*. Unlike other nidoviruses, mesoniviruses are considered insect-specific viruses (ISVs) mainly found in mosquitoes and are not known to infect vertebrate cells. This family contains only one subfamily, *Hexponivirinae*, that currently contains a single genus*—Alphamesonivirus*—and nine subgenera (https://ictv.global/taxonomy).

The genome of Alphamesonivirus-1 (*Alphamesonivirus cavallyense*, subgenus *Namcalivirus*) contains seven open reading frames (ORFs), with ORF1a and -1b located at the 5′ end, encompassing two-thirds of the genome, and the smaller ORF2a, -2b, -3a, -3b, and -4 occupying the 3′-end of the genome. The five major 3′-ORFs are predicted to encode a spike (S) glycoprotein (in ORF2a), a nucleocapsid (N) protein (in ORF2b), two proteins with membrane-spanning regions (in ORF3a and -3b), and a small protein with unknown function (in ORF4) (8, 9).

Alphamesonivirus-1 comprises most of the mesoniviruses identified to date and was initially considered the prototype species of the family (https://ictv.global/taxonomy). The family was first represented by two closely related viruses: Cavally virus (CavV), isolated in Ivory Coast and initially named as a first insect-associated nidovirus (8), and Nam Dinh virus (NDiV) isolated in Vietnam (10), with the new family *Mesoniviridae* proposed the following year (11). To date, mesoniviruses have only been identified from naturally infected mosquitoes (12, 13), with no reports in vertebrates (14, 15), and hence are considered ISVs (16, 17) in a similar manner to insect-specific flaviviruses (14) and mosquito-associated bunyaviruses (18). Of note, a growing number of mesonivirus species have been identified from mosquitos collected in the Americas (19), Asia (20), Africa (12), and Australia (21), suggesting a near global distribution. Additionally, a mesonivirus was identified from *Aphis citricidus* aphids collected in China in 2012 (22), while a mesoni-like virus has been detected in an obligate fungal pathogen*—Leveillula taurica*—in Italy (17). Hence, the host range of mesoniviruses is likely to be broader than currently known.

Here, using a combination of genomic and histological techniques, we identified Alphamesonivirus-1 in two horses that succumbed to an acute respiratory syndrome. This represents the first detection of a mesonivirus in a vertebrate.

## RESULTS

### Bronchopneumonia and unspecific-viral infection detected in the lungs and lymph nodes of two horses that succumbed to an acute respiratory syndrome

**Fig 1:**
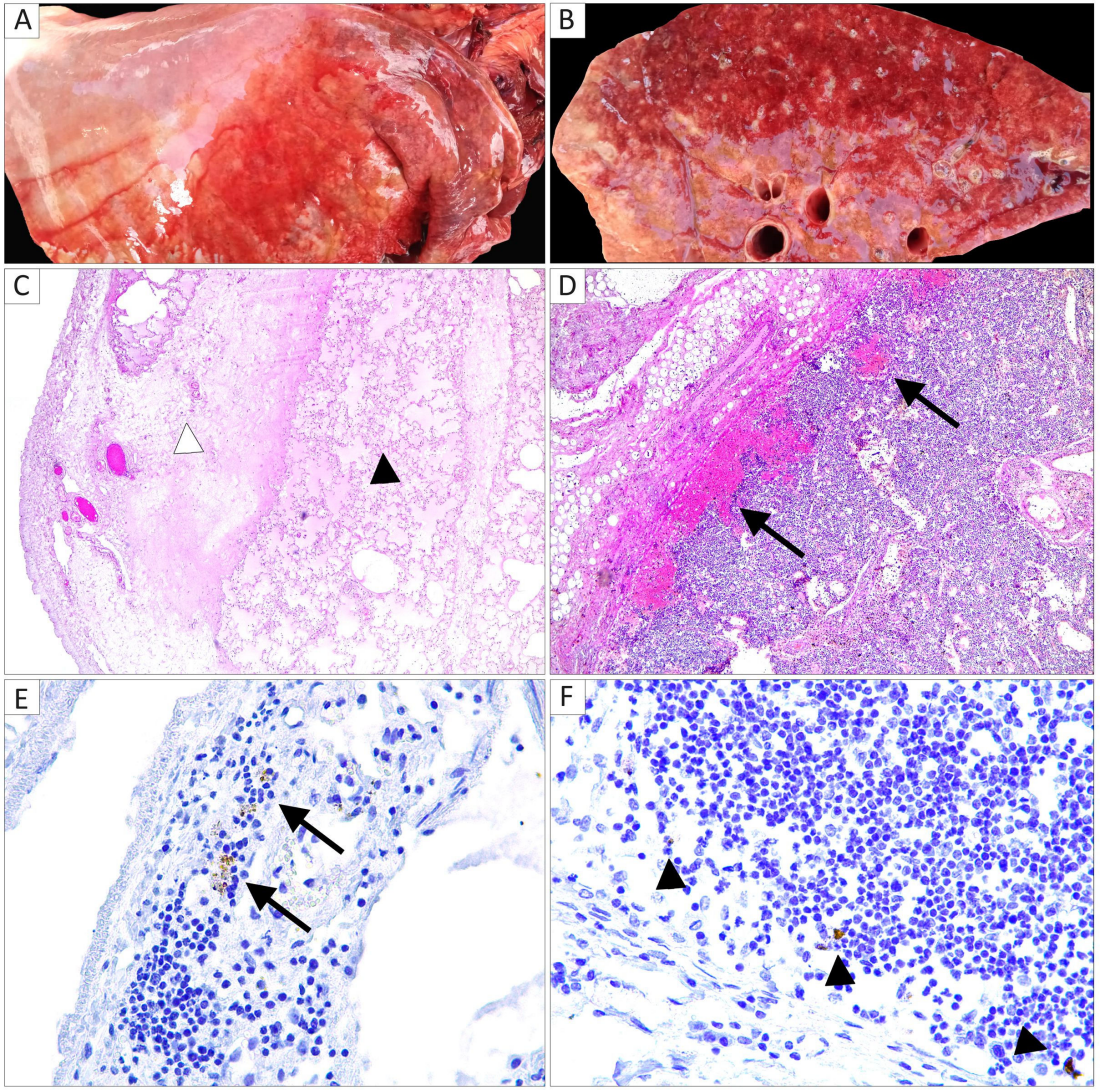
Organ photographs and histological staining of tissues from the horses that succumbed to an acute respiratory syndrome. On gross examination of the lung, the mare showed pleural effusion (A), and on cut section, pulmonary oedema and marked enlargement of interlobular septa were apparent (B). Histological analyses (hematoxylin and eosin stain) in the lung demonstrated (C; 50× magnification) thickness of visceral pleura (black arrowhead) and diffuse alveolar oedema (white arrowhead), and in a bronchial lymph node (D; 100× magnification) sub-cortical multifocal hemorrhages (black arrows). Alphamesonivirus-1 *in situ* hybridization (400× magnification) detected intracytoplasmic signals visualed as brown spots in scattered mononuclear cells compatible with macrophages residing in a lung alveolar septum (E, black arrows) and in the sub-capsular sinus of a bronchial lymph node (F, black arrowheads).

The body condition score of the horse carcasses was in the physiological range, with no signs of external trauma observed. The mare was in foal, and the intra-uterus fetus was of normal physiological size and shape. Advanced putrefaction and decomposition of internal digestive organs as late post-mortem changes were apparent. Post-mortem analysis revealed foamy nasal discharge, severe gelatinous subcutaneous oedema of the neck region, and hemorrhagic pleural exudate, while severe pulmonary oedema and thickness of interlobular septa were observed in the lungs of both horses (Fig. 1A and B). Splenomegaly, enlargement of bronchial, submandibular, and retropharyngeal lymph nodes (LN), and petechial hemorrhages of the large intestine mucosa were evident as well. Histopathology analyses revealed severe alveolar oedema, massive and severe thickness of visceral pleura, areas of bronchopneumonia and hemorrhagic foci in bronchial, submandibular and retropharyngeal lymph nodes (Fig. 1C and D). Both the mare and the foal showed gross and microscopic similar lesions in terms of localization, type, and extension (Fig. 1C and D).

All samples tested negative for the pathogens for which direct diagnostic tests were available, including EHV1, EHV4, WNV, USUV, EAV, IAV, AHSV, *Babesia caballi*, *Theileria equi*, and *Trichinella spiralis* (Table 1). Bacterial growth was not observed when the brain, lung, and lymph nodes homogenates were cultured with standard protocols. Anaerobic bacterial growth was observed in spleen, kidney, and liver tissue homogenates likely as result of post-mortem proliferation.

**TABLE 1:** Pathogen detection in horses (mare and foal) and a *Culex* mosquito pool^*a*^^,^^*d*^

Pathogen	Method	Horse samples^*b*^	Results		
			Mare	Foal	*Culex* mosquito pool^*c*^
Equine herpesvirus 1 and 4(EHV1 and EHV4)	EHV1/4-specific real-time RT-PCR	Retropharyngeal, submandibular, and bronchial lymph nodes, lungs, spleen	Negative	Negative	Not tested
West Nile virus (WNV)	WNV-specific real-time RT-PCR		Negative	Negative	Not tested
Usutu virus (USUV)	USUV-specific real-time RT-PCR		Negative	Negative	Not tested
Equine arteritis virus (EAV)	EAV-specific real-time RT-PCR		Negative	Negative	Not tested
Influenza A virus (IAV)	IAV-specific real-time RT-PCR		Negative	Negative	Not tested
African horse sickness virus (AHSV)	AHSV-specific real-time RT-PCR		Negative	Negative	Not tested
*Babesia caballi*	*Babesia caballi* conventional PCR	Whole blood	Negative	Negative	Not tested
*Theileria equi*	*Theileria equi* conventional PCR	Whole blood	Negative	Negative	Not tested
*Trichinella spiralis*	Magnetic stirrer method	Diaphragmatic muscle	Negative	Negative	Not tested
Alphamesonivirus-1	Pan-Mesonivirus real-time PCR^d^	Lungs	Positive (CT = 34)	Positive (CT = 33)	Not tested
Alphamesonivirus-1	*In situ* hybridization	Lungs	Positive	Positive	Not tested
Non-targeted	Metatranscriptomic screening	Lungs	Alphamesonivirus-1, *Massilia* sp., *Bradyrhizobium* sp., *Bacillus* sp., *Filimonas* sp., *Chitinophaga* sp., *Providencia* sp.	Alphamesonivirus-1, Biggie virus *Bradyrhizobium* sp., *Panacibacter* sp., *Filimonas* sp., *Chitinophaga* sp., *Escherichia* sp.	Alphamesonivirus-1, Tombus-like virus, *Wolbachia* ap., Biggie virus

^*a*^ Results are only shown for the one pool found positive for Alphamesonivirus-1 using a metatranscriptomic approach.

^*b*^ The supernatant of homogenized organ samples or whole blood was used for the horse samples.

^*c*^ The supernatant of the homogenized mosquito pool was used for the metatranscriptomic analysis of the *Culex* pool.

^*d*^ Performed on double-stranded cDNA obtained after SISPA protocol.

### Identification and confirmation of Alphamesonivirus-1 in horse samples

Metatranscriptomic sequencing produced a total number of 28,231,914 and 26,934,576 raw reads from the foal and mare lung tissue samples, respectively. After quality checks and trimming, the remaining 26,944,930 and 25,958,036 reads were analyzed using the CZID tool: this assigned 14,683 reads (0.73% of all reads, foal) and 746 (0.43%, mare) reads to Alphamesonivirus-1 strain pool 11/2008 (GenBank MF281710.1) identified from a *C. pipiens* pool in north Italy in 2008. Similar results were obtained by sequential Blast analyses, with RPKM values for Alphamesonivirus-1 ranging from 28.47 to 54.48 for the foal, and 0.96–1.63 for the mare. Hence, the virus was at considerably higher abundance in the foal than the mare.

A subsequent reference-based assembly of Alphamesonivirus-1 by iVar (1.3.1) produced two consensus sequences with a horizontal coverage (Hcov) of 99% for the foal sample and 57% for the mare sample. Hcov for the mare sample was improved to 87% after re-mapping of reads using the Alphamesonivirus-1 consensus sequence obtained from the foal sample as reference. Mean vertical coverage (Vcov) was 37.22 (min = 1, max = 554) for the foal sample and 5 (min = 0, max = 65) for the mare sample. Pan-mesonivirus molecular assay confirmed the presence of RNA belonging to Alphamesonivirus-1 in both samples and threshold cycles (Ct) were 33 and 34 for foal and mare lungs, respectively.

In addition to the equine samples, 10 pools of *Culex* sp. were analyzed using this metatranscriptomic protocol. The taxonomic classification of reads by CZID revealed the presence of reads assigned to Alphamesonivirus-1 species in one pooled sample collected in Sant’Omero municipality (Teramo province, Abruzzo region, a neighboring region of Molise). Deep sequencing of this sample resulted in 30,775,924 raw reads. After quality checks and trimming, the remaining 13,125,482 reads were again analyzed using CZID. As before, the most abundant species was Alphamesonivirus-1 (15.07%, with 1,977,945 reads), followed by arthropod-specific microbial species including *Culex*-associated Tombus-like virus (8.7%), *Wolbachia* bacteria (1.53%), and a member of the *Negevirus* genus of positive-sense RNA viruses (0.23%; see below).

*In situ* hybridization for *Alphamesonivirus-1* in both animals demonstrated intracytoplasmic occurrence of viral RNA in mononuclear cells morphologically compatible with macrophages residing in lung alveoli and in the sub-capsular sinus of a bronchial lymph node (Fig. 1E and F).

### Presence of additional microbial species

As noted above, as well as Alphamesonivirus-1, a variety of other microbial species were identified in the horse samples following metatranscriptomics analysis. Perhaps of most note was the presence of transcripts for Biggie virus (*Negevirus*, unclassified positive-sense RNA virus) in the foal (RPKM = 1.63), matching its identification in the *Culex* samples.

In addition, almost 2 million reads from the foal lung sample and 250,000 reads from the mare lung samples were assigned to different bacterial species known to be normally non-pathogenic or environmental contaminants (Table 1). Reads assigned to the *Clostridium* genus represented the highest relative sequence abundance in both the mare (91.09%) and the foal (87.27%). Although the vast majority of clostridial species are non-pathogenic commensal or soil bacteria, *Clostridium perfringens* and *C. botulinum* are responsible for severe diseases of horses (23). Therefore, mapping analysis was performed using the reference sequence for *C. perfringens* (GenBank CP009557.1) and for *C. botulinum* (CP063816.1) as indicated by the CZID tool. Mapping analysis for both samples failed, resulting in Hcov of 0.01% (foal) and 0.04% (mare) for *C. perfringens* and Hcov of 0.04% (foal) and 0.03% (mare) for *C. botulinum*.

### Characterization of horse Alphamesonivirus-1

**Fig 2:**
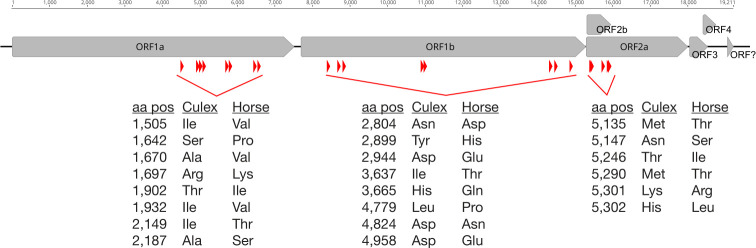
Amino acid substitutions that distinguish the Alphamesonivirus-1 sequences obtained from the horses and from the mosquito pool. A total of 22 amino acid mutations were identified (red arrows) between the Alphmesonivirus-1 sequence from the *Culex* mosquito pool collected in 2022 from the Abruzzo region of Italy and the sequence obtained from the horses with acute respiratory syndrome sampled in 2021. A schematic of the Alphmesonivirus-1 genome is shown.

**Fig 3:**
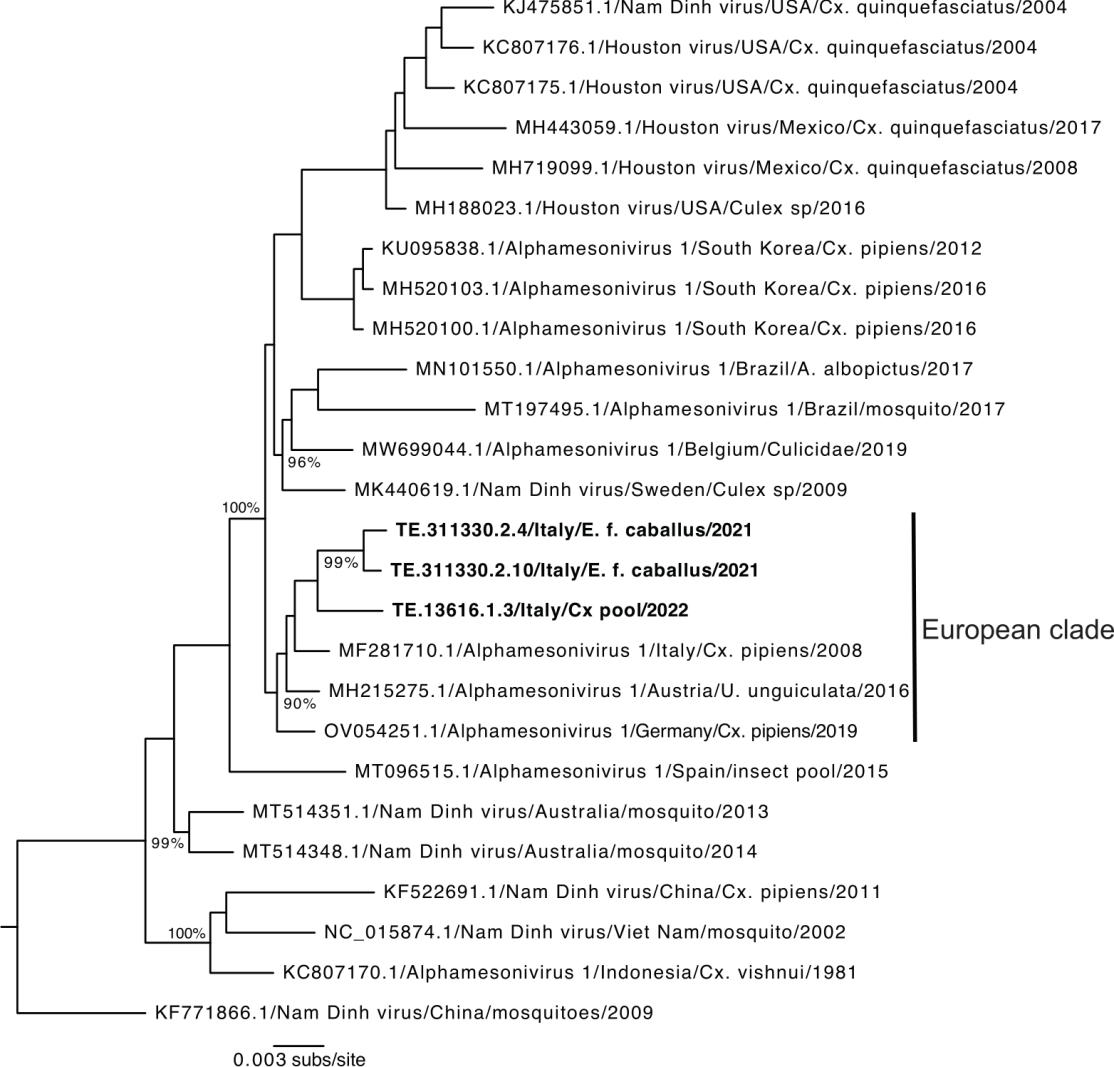
Phylogenetic analysis of the alphamesoniviruses showing the position of the Italian sequences. Maximum likelihood tree of the full genome sequences of alphamesoniviruses with the Alphamesonivirus-1 sequences generated in this study marked in bold (E. f. caballus = horse sequences; Cx pool = mosquito sequences). The European clade of sequences is also marked. The tree is rooted on the first detected Nam Dinh virus sequence (from 2009), and all horizontal branches are drawn to a scale of nucleotide substitutions per site. Bootstrap support values >70% are shown at main internal nodes.

The Alphamesonivirus-1 sequences obtained from the foal and mare samples were identical with the exception of ambiguities due to low coverage (resulting in an overall pairwise nt identity of 98.5%). A total of 22 amino acid substitutions were found between the Alphamesonivirus-1 sequences obtained from the horses and from the *Culex* mosquito pool (Fig. 2). Overall, eight non-synonymous mutations were found in ORF1a, and ORF1b each, and six in ORF2a. A phylogenetic tree of Alphamesonivirus-1 was estimated using those sequences generated here combined with publicly available complete genome sequences (Fig. 3). This revealed some geographical clustering, with monophyletic groups for virus sequences sampled from South Korea, North America, Australia, and Asia. Notably, the sequences generated in this study formed a well-supported monophyletic group (99% bootstrap support) within a European clade (90% bootstrap support) that includes an Alphamesonivirus-1 sequence identified in Italy in 2008 which falls as the sister-group to the horse and *Culex* pool sequences. Hence, this topological pattern is indicative of the ongoing transmission of Alphamesonivirus-1 in Italy.

There was no evidence for molecular clock structure in these data (negative correlation coefficient −0.29, *R* squared 0.08), suggesting that Alphamesonivirus-1 is evolving relatively slowly over the time course of sampling and precluding more detailed molecular clock analysis. This is in marked contrast to other *Nidovirales* such as SARS-CoV and SARS-CoV-2 that have experienced measurable evolutionary rates of ~10^−3^ nucleotide substitutions per site, per year during their spread through human populations (24). Although some sporadic *in silico* evidence for limited positive selection across the Alphamesonivirus-1 phylogeny was observed, there was no evidence for any adaptive evolution associated with the Italian viruses (results not shown).

## DISCUSSION

Respiratory infections are important causes of morbidity and mortality in horses. However, the causative agents are frequently unidentified, often misdiagnosed, or overlooked. Infections of the upper respiratory tract, both viral and bacterial, are usually diagnosed in weanling and yearling horses, while airway disorders, pleuropneumonia, or epistaxis conditions caused by inflammation or exercise are found in horses older than two years. In contrast, recurrent airway disease or neoplasia of the respiratory tract is diagnosed primarily in middle-aged and older horses (3). Conditions such as inflammatory airway disease, chronic obstructive pulmonary disease, or exercise-induced pulmonary hemorrhage are examples of the pathological conditions affecting the respiratory system of horses whose complex etiology remains uncertain.

Mesoniviruses have no known association with animal disease, and to the best of our knowledge, this is the first report of a mesonivirus in a vertebrate. The presence of Alphamesonivirus-1 in two horses located in the same barn was indicated by metatranscriptomic analysis and confirmed by an Alphamesonivirus-1-specific-PCR and *in situ* hybridization on infected pulmonary tissues. Phylogenetic analysis revealed that the Alphamesonivirus-1 identified from the mare and the foal from the Molise region of Italy were closely related to a sequence derived from a mosquito pool collected in the neighboring Abruzzo region in 2022. The high genetic similarity between these sequences, and to sequence from a virus identified in north Italy in 2008, suggests that Alphamesonivirus-1 is continuously circulating in Italy. However, additional work is required to determine whether the presence of Alphamesonivirus-1 in these two horses was indicative of a larger scale infection in equine populations or merely a transient spill-over event that died out with no additional infections. If the former, then the more widespread metagenomic screening for mesoniviruses in mammalian populations is warranted. Similarly, it is currently uncertain whether the virus was maternally transmitted from mare to foal, or whether these animals were simultaneously infected by local *Culex* mosquitoes.

Microbiological analyses of kidneys, liver, and spleen provided positive results for the presence of anaerobic bacteria. However, culture isolation, including the pathogenic *Clostridium perfrigens*, yielded negative results. As such, the anaerobic bacterial growth observed was likely due to post-mortem changes in the decomposition and putrefaction of the internal organs.

As no other recognized horse pathogens were identified in our analysis, the possible role played by Alphamesonivirus-1 infection in the etiology of the acute respiratory syndromes observed here, either alone or in association with another infectious agent, should be considered. Additional experimental investigation is therefore warranted, particularly as the mechanisms triggering any infection remain unknown. Indeed, the low viral load found in our study indicated by high Ct values and the low number of Alphamesonivirus-1 reads in the mare (although not in the foal) calls into the question whether this virus was the causative agent of the fatal respiratory disease in the horses. In this context, it is noteworthy that similar circumstances were observed with the initial detection of Schmallenberg virus in blood samples from cows (25). Schmallenberg virus is transmitted by the female biting midges of the *Culicoides obsoletus* complex and is associated with disease in ruminants including fever, fetal malformation, drop in milk production, diarrhea, and stillbirths, becoming a burden for small and large farms (26). In addition, there is increasing evidence that groups of RNA viruses normally assumed to be insect specific, such as jingchuviruses can in fact replicate and reach relatively high abundance in mammalian tissues (27), with discistroviruses also present in the serum and plasma of febrile patients (28). The possibility that ISVs may, in fact, sporadically infect vertebrates should therefore be given greater attention.

We similarly cannot exclude that a cytokine storm following Alphamesonivirus-1 infection played a major role in the observed respiratory syndrome, particularly as this is well documented in related *Nidovirales* including feline infectious peritonitis virus and SARS-CoV-2 (29–32). COVID-19 pathology was generally characterized as biphasic with an acute phase dominated by active SARS-CoV-2 infection and a post-viral clearance phase dominated by host reparative and immunologic processes (33). Hence, since macrophages in the sub-capsular sinus in a bronchial lymph node and in lung alveoli were shown to be positive for Alphamesonivirus-1 by *in situ* hybridization, we cannot exclude macrophage hyperactivation in the two horses. This scenario is also described in SARS-CoV-2 infected individuals in which CD169+ macrophages were detected in lymph node subcapsular spaces (34). Macrophage disorders such as secondary hemophagocytic lymphohistiocytosis have been well described in COVID-19 and in other coronavirus infections, including SARS-CoV and MERS-CoV (35). Hemophagocytic lymphohistiocytosis is a hyperinflammatory syndrome characterized by a fulminant and fatal hypercytokinaemia with multiorgan failure in humans. In adults, this phenomenon is mostly triggered by viral infections, autoimmune diseases, and neoplasms (36).

The presence of the Biggie virus (*Negevirus*) in the foal and in the *Culex* mosquito pool was also intriguing although virus abundance was very low in the former. Biggie virus is associated with *Culex* mosquitoes and was first identified at relatively high abundance in *C. pipiens* and *C. torrentium* from Sweden (37). Although there is no prior evidence for negeviruses in mammalian species, Alphamesonivirus-1 and negeviruses were recently found to interact *in vitro* with several co-infecting arboviruses (*Flaviviridae*, *Togaviridae*, *Peribunyaviridae*) inhibiting USUV and Bunyamwera orthobunyavirus infection (38). Whether the co-infection of Alphamesonivirus-1 and Biggie virus observed in the foal was simply a chance occurrence, or represents a form of functional interaction, merits additional attention, as does whether this co-infection impacts disease pathology and/or inhibits the presence of other viruses.

This study has a number of limitations. Despite the use of different mammalian and mosquito cell lines (VeroE6 and C6/36), we were unable to isolate the virus. As the experiments undertaken by Diagne et al. (12) demonstrated the ability of Alphamesonivirus-1 to replicate in the mosquito C6/36 only at 28°C, but not at 37°C, we performed all isolation attempts (including with mammalian cells) at 28°C. Another limitation was that we were unable to perform a serological screen of healthy horses from the same farm to investigate the presence and prevalence of Alphamesonivirus-1 in the remainder of the herd. Due to the lack of specific serological tools for Alphamesonivirus-1, local virus circulation and potential seroconversion in horses could not be addressed. In addition, it would have been beneficial to collect mosquitoes from the same farm. Finally, due to disease presentation as an acute respiratory syndrome, the gross lesions described, and the advanced state of putrefaction observed in the carcasses, we initially focused only on the lungs and did not perform the Alphamesonivirus-1 PCR and metatranscriptomics on other organ samples.

The identification of Alphamesonivirus-1 in two horses enhances our understanding of the diversity and biology of mesoniviruses and may provide new insights into the pathogenesis of respiratory diseases of horses. The presence of Alphamesonivirus-1 in the lungs of the horses studied here is an important first step in understanding mesonivirus host range, evolution, and its potential to infect and potentially cause disease in other animals than insects. Since mesoniviruses have not previously been reported in mammalian hosts, our identification of a supposedly insect-specific virus in a mammalian host necessitates additional *in vivo* investigation and broader surveillance of this virus.

## MATERIALS AND METHODS

### Sample collection and diagnostic approach

In October 2021, an 18-month-old foal and a 7-years-old mare Haflinger horse died unexpectedly due to acute respiratory syndrome in the same farm located in Miranda, province of Isernia, Molise region, Italy. The two carcasses were sent to the *Istituto Zooprofilattico Sperimentale dell’Abruzzo e del Molise* (IZSAM) for necropsy. Lung and bronchial lymph nodes were sampled from each animal, fixed in 10% neutral buffered formalin, routinely processed for histology, and stained with Haematoxylin and Eosin (HE). Samples from the retropharyngeal, submandibular, and bronchial lymph nodes, lungs, and spleens were collected and homogenized in a sterile phosphate-buffered saline (PBS) and then centrifuged. Nucleic acid was extracted from 200 µL of supernatants using the MagMAX CORE Nucleic Acid Purification Kit (Applied Biosystems) on an automatic extractor KingFisher Flex (ThermoFisher Scientific), with an elution volume of 100 µL, following the manufacturer’s instructions. The samples were tested by molecular assays for the presence of RNA/DNA pathogens for which direct diagnostic tests were available, including EHV1 and EHV4 (39), West Nile virus (WNV) (40), Usutu virus (USUV) (41), *Alphaarterivirus equid* (EAV) (VetMax EAV Kit, Applied Biosystems), IAV (42, 43), African horse sickness virus (AHSV) (44). Whole blood samples were analyzed for the detection of *Babesia caballi* and *Theileria equi* by conventional PCR (45, 46), while diaphragmatic muscle was tested for the presence of *Trichinella spiralis* by means of magnetic stirrer method (47). All samples were also tested by standard procedures for aerobic and anaerobic bacterial culture.

### Sample preparation, library construction, and metatranscriptomic sequencing

To assist with pathogen identification, RNA purified from lung samples of both horses was processed for metatranscriptomic analysis. After Turbo DNAse (Thermo Fisher Scientific, Waltham, MA, USA) treatment at 37°C for 20 min, total RNA was purified by an RNA Clean & Concentrator−5 Kit (Zymo Research, Irvine, CA, USA). The RNA obtained was processed using sequence-independent single-primer amplification protocol (SISPA) with some modifications (48). The amplicons were purified by ExpinTM PCR SV (GeneAll Biotechnology CO., LTD Seoul, Korea) and quantified by Qubit dsDNA HS assay (Thermo Fisher Scientific, Waltham, MA, USA). The samples were diluted to obtain a concentration of 100–500 ng and used for library preparation with the Illumina DNA Prep kit (Illumina Inc., San Diego, CA, USA) according to the manufacturer’s protocol. Deep sequencing was performed on the NextSeq 500 (Illumina Inc., San Diego, CA, USA) using the NextSeq 500/550 Mid Output Reagent Cartridge v2, performing 300 cycles, and generating 150 bp paired end reads. Raw sequencing reads underwent quality trimming before adapter removal using Trimmomatic v0.38. Quality control of raw and trimmed reads was performed with FASTQC v0.11.8.

Fastq files were initially analyzed using the Chan Zuckerberg ID (CZID) software (https://czid.org/), an open-source software platform that helps identify pathogens in metatranscriptomic sequencing data after host sequence removal. Following indications on microbial composition provided by CZID, fastq data of both horses were mapped by the BWA software package (v.0.7.17) (49) to Alphamesonivirus-1 reference accession number NC_015668. Alphamesonivirus-1 consensus sequences were obtained by iVar (v1.3.1). Paired-end reads were *de novo* assembled into contigs using MEGAHIT v1.2.9 with default settings. To confirm these initial observations, the assembled contigs were compared to the NCBI non-redundant database (NCBI-nr) using DIAMOND v2.1.6 with an e-value cut-off ≥1E−4 (50). To provide further validation of hits, contigs were screened against the nucleotide database (NCBI-nt) with an *e*-value cut-off ≥1E−10. Virus abundance was quantified and normalized by contig length using TPM (transcripts per million) and RPKM (reads per kilobase million) metrics as implemented in RSEM v1.3.0.

Through genomic surveillance activities performed at IZSAM in 2022 within the National surveillance system of arboviral diseases, 10 pools of *Culex* sp. were collected in different parts of Teramo province, Italy (in the Abruzzo region, a neighboring region to Molise). These samples also underwent metatranscriptomic analysis using the protocol described above.

### Specific Pan-Mesonivirus molecular assay

The presence of Alphamesonivirus-1 was confirmed by real-time PCR assay using primers and probes as described in Diagne et al. (12). PCRs were prepared with 10× GoTaq Probe qPCRMaster Mix (Promega) containing final concentrations of 0.5 mM forward primer, 0.5 mM reverse primer, 0.25 mM TaqMan probe, 5 µL of double-stranded cDNA obtained after SISPA protocol, and nuclease-free water up to 20 µL reaction volume. Real-time PCR reactions were performed on a QuantStudio 7 Flex Real-Time PCR System (Applied Biosystems) in fast mode with the following settings: initial denaturation at 95°C for 20 s, followed by 40 cycles of denaturation at 95°C for 1 s and annealing/extension at 55°C for 20 s.

### RNA *in situ* hybridization

Lung and bronchial lymph nodes derived from the foal and mare were subjected to an RNA *in situ* hybridization performed by RNA scope analysis platform (RNAscope 2.5 HD Assay – BROWN kit, Biotechne) following the manufacturer’s instructions. To detect viral RNA, sections were incubated with an *ad hoc* probe designed and manufactured commercially (ACDbio, Bio-Techne, USA). The probe was designed to detect Alphamesonivirus ORF1a (GenBank MT096515.1) targeting nt 1084–2098. The endogenous housekeeping gene Ubiquitin C (UBC) was used as positive control to assess both tissue RNA integrity and assay procedure. Slides were counter-stained with Mayer’s Hematoxylin (Bio-Optica, Italy) and mounted with Eukitt mounting media (Bio-Optica, Italy) before analyzing on a Zeiss Axio Scope.A1 microscope (Carl Zeiss Microscopy GmbH, Göttingen, Germany).

### Phylogenetic and evolutionary analysis

Nucleotide sequences representing the full genome of Alphamesonivirus-1 were obtained from NCBI GenBank (accessed 5 June 2024; *n* = 52) and combined with two reference sequences (NC_015874.1 and NC_015668.1) and the two sequences isolated from both horses and from the mosquito pool. A multiple sequence alignment was performed in Mafft v7.450 using the FFT-NS-i x1000 algorithm (51). The alignment was manually inspected in Geneious Prime 2021.1.1 (https://www.geneious.com) for accuracy. Sequences with extensive genetic diversity were removed. A maximum likelihood (ML) phylogenetic tree was then estimated for the final data set of the selected full-genome sequences using RAxML v 8.2.11 (52) implementing a gamma time reversible + Γ model of among suite rate heterogeneity (GTR+ Γ) nucleotide substitution model and 200 bootstrap replicates.

To assess the extent of temporal (i.e., clock-like) structure in the data, we performed a regression of root-to-tip genetic distance on the ML tree against date (year) of sampling using the TempEST method (53). The lack of temporal structure (see Results section) precluded additional analyses of evolutionary rates or time scales.

To test for the presence of positive selection (i.e., adaptive evolution), especially involving the amino acid substitutions associated with the viruses in the horses, we utilized the FUBAR (54), MEME (55), BUSTED (56) methods in HyPhy/Datamonkey (57, 58) that explore various distributions of the numbers of nonsynonymous (*d*_N_) and synonymous (*d*_S_) substitutions per site.

## Data Availability

The two Alphamesonivirus-1 consensus sequences obtained in this study from the Haflinger mare and foal, as well as the sequence from the *Culex* mosquito pool, were deposited in the NCBI/GenBank database under accession numbers PP961236, PP961235, and PP961237, respectively. Raw metatranscriptomic data are available in the Sequence Read Archive (SRA) under BioProject number PRJNA1126112.
